# Non-Invasive Human-Free Diagnosis Methods for Assessing Pig Welfare at Abattoirs: A Systematic Review

**DOI:** 10.3390/ani15172500

**Published:** 2025-08-25

**Authors:** Maria Francisca Ferreira, Márcia Nunes, Madalena Vieira-Pinto

**Affiliations:** 1 CECAV—Veterinary and Animal Research Centre, Department of Veterinary Sciences, University of Trás-os-Montes and Alto Douro (UTAD), 5001-801 Vila Real, Portugal; 2 School of Agrarian and Veterinary Sciences (ECAV), Department of Veterinary Sciences, University of Trás-os-Montes and Alto Douro (UTAD), 5001-801 Vila Real, Portugal; 3 Associate Laboratory for Animal and Veterinary Sciences (AL4AnimalS), 5001-801 Vila Real, Portugal

**Keywords:** swine, indicators, abattoir, tools, health, risk-based meat inspection

## Abstract

The assessment of pig welfare and health at abattoirs is crucial for ensuring both animal well-being and food safety. Traditional assessment methods often rely on human observation, which is time-consuming, subjective, and difficult to scale in high-throughput facilities. This systematic review addresses a crucial gap by identifying and evaluating non-invasive human-free diagnostic methods applicable in commercial settings. Following PRISMA guidelines, a total of 102 articles met the inclusion criteria. Thirteen distinct methods were identified and classified into three categories: biological sample analysis (5 methods; *n* = 80 articles), imaging and computer vision systems (4 methods; *n* = 19), and physiological and other sensors (4 methods; *n* = 24). Some articles assessed more than one method and are therefore counted in multiple categories. While no method achieved both high implementation and practicality, blood analysis for glucose and lactate, convolutional neural networks for lesion detection, and automated camera-based systems emerged as the most promising for practical integration into the slaughter flowline. Most techniques still face challenges related to automation, operator independence, and standardisation. Overall, this review highlights the growing potential of non-invasive methods in pig welfare evaluation and underscores the need for continued development and validation to facilitate their adoption into routine abattoir practices.

## Simple Summary

Slaughterhouses serve as critical checkpoints for monitoring pig welfare and health, as they facilitate the evaluation of pre-slaughter handling and enable post-mortem health assessments. Ensuring both animal welfare and food safety is central to these evaluations. This review examines non-invasive human-free methods for assessing pigs without physical restraint or subjective human interpretation, thereby minimising stress while enhancing objectivity. Thirteen categories of tools were identified, including thermal imaging to monitor stress-related temperature changes and artificial intelligence-driven imaging for automated lesion detection. Other diagnosis methods, such as blood analysis at exsanguination, provide valuable indicators without compromising animal well-being. However, their widespread adoption faces challenges related to feasibility, standardisation, and integration into high-speed slaughter lines with real-time monitoring. Ultimately, these tools can support risk-based meat inspection, benefiting both the industry and consumer expectations.

## 1. Introduction

Monitoring pig welfare and health at abattoirs is essential for ethically responsible meat production, food safety, and public health, as well as for meeting the increasing consumer demand for high animal welfare standards. Abattoirs serve as critical checkpoints to evaluate the impact of transport and pre-slaughter handling on pig welfare and to detect health conditions that may result in substantial economic losses, such as carcass downgrading and reduced meat quality [1,2].

Within the European Union, abattoirs processing more than 1000 animal units per year are legally required to document animal welfare practices [3]. Compliance with these regulations is the responsibility of the animal welfare officer (AWO), who implements corrective action plans where necessary. Simultaneously, the official veterinarian (OV) oversees official controls, including ante-mortem and post-mortem inspections [4], and verifies food chain information [5]. However, high-throughput abattoirs present significant challenges due to the demanding workload per inspector, which can lead to fatigue and reduced attention to detail. Additionally, variability in human assessment methods can result in low inter-observer reliability, complicating the standardisation of welfare evaluations [6].

These constraints underscore the need to develop and implement rapid, reliable, and systematic tools for monitoring welfare and health indicators in high-throughput abattoirs [7]. Such tools must be non-invasive to avoid compromising animal welfare and must ensure objectivity and accuracy. Technologies such as thermal imaging, biosensors, and automated behavioural monitoring allow diagnosis without physical restraint or subjective human interpretation. While existing reviews have addressed animal welfare indicators [8], this systematic review aims to fill a key gap by providing a practical and concise overview of non-invasive human-independent diagnostic tools already evaluated in commercial abattoirs.

Beyond welfare assessment, technological advances in automated systems have shown promise for improving post-mortem health evaluations [6,7,9]. Tools based on automated imaging and artificial intelligence can provide objective assessments, enhance lesion detection, reduce observer bias, and minimise the risk of cross-contamination between carcasses [9,10]. Moreover, they enable systematic data collection, facilitating easier processing and analysis. These approaches align with modern risk-based meat inspection strategies, which prioritise public health hazards and improve the efficiency of official veterinary controls [11].

This systematic review was guided by the following research question: “What human-independent and non-invasive diagnostic methods are currently available to assess the welfare of slaughtered pigs at both ante-mortem and post-mortem stages?”

## 2. Methodology

### 2.1. Information Sources and Search Strategy

The design and reporting of this systematic review followed the guidelines outlined in the preferred reporting items for systematic reviews and meta-analyses (PRISMA) statement [12].

The initial pool of articles was primarily established by re-screening the 227 articles identified in a recent systematic review by Huanca-Marca et al. [8]. That review focused on validated indicators for assessing pig welfare at slaughter, providing a comprehensive foundation of the relevant literature. The eligibility of these 227 articles was re-evaluated against the specific inclusion and exclusion criteria applied in the present review (detailed in Section 2.2).

To ensure comprehensive coverage of all non-invasive human-independent diagnostic tools, a complementary systematic search was conducted. Three databases—PubMed, Scopus, and ScienceDirect—were systematically searched for articles published between 2000 and October 2024. Only studies published in English, Portuguese, or Spanish were considered. The search strategy was tailored to the objectives of the current review and employed a combination of keywords and Boolean operators: (pig OR swine) AND (abattoir OR slaughterhouse) AND (welfare) AND (non-invasive OR technology OR image OR thermography OR vision OR artificial intelligence). The grey literature was excluded to ensure that only peer-reviewed and formally published studies were considered.

### 2.2. Eligibility Criteria

This review focused exclusively on methods that utilise external or minimally invasive approaches, thereby excluding methods that involve puncture-based or tissue extraction methods on live pigs.

Articles were included if they were primary studies, presenting original data, and published in peer-reviewed scientific journals. These studies focused on pigs evaluated at any stage within a commercial abattoir environment from unloading to post-mortem inspection. The diagnostic method utilised had to be non-invasive and human-independent. This included tools that do not require direct human intervention or assessment, such as thermal imaging or computer vision systems. It also included methods in which indicators, for example, those derived from blood, saliva, or urine, could be assessed without subjective human interpretation.

Conversely, studies were excluded if they did not report peer-reviewed and primary data. We also excluded studies primarily focused on on-farm welfare, transport, or meat quality, unless they specifically presented relevant data from abattoir stages using eligible methods. Furthermore, any studies that used invasive tools (e.g., methods involving puncture on live animals) to assess pig welfare were excluded. Exclusion was also applied to studies where indicators were assessed solely through direct human observation or if the diagnostic method used was not clearly described. Studies conducted in research plant settings were also excluded.

### 2.3. Relevance Screening

All 227 articles identified from Huanca-Marca et al. [8] and those retrieved from the complementary search were imported into the Rayyan platform (https://www.rayyan.ai, accessed on 5 January 2025). Duplicates were subsequently removed.

A multi-stage screening process was conducted to minimise the risk of bias. Initially, a single researcher (MFF) performed a preliminary screening of each article’s title, abstract, and keywords against the eligibility criteria, utilising Rayyan’s screening form. Subsequently, two independent researchers (MMVP and MN) re-evaluated the full set of articles based on their titles and abstracts. All screening at this stage was conducted independently, with researchers blinded to each other’s responses. Any disagreements between the reviewers were resolved through discussion and consensus.

Following this initial relevance confirmation, a team member (MFF) retrieved and screened the full-text documents of all potentially eligible articles for final inclusion. Reasons for exclusion at the full-text stage were recorded.

### 2.4. Data Extraction

Data extraction for descriptive analysis was performed using Microsoft Office Excel^®^ 2016 (Microsoft Corporation, Redmond, WA, USA). A standardised data extraction form was developed and applied to all included articles. The following key information was systematically extracted: publication details, the diagnostic tool used, and the indicators measured or detected by the tool.

### 2.5. Classification of the Findings

Data were categorised into three groups: biological sample analysis, imaging and computer vision systems, and physiological and other sensors. They are presented to provide a practical and straightforward overview of the identified methods. The synthesis specifically addresses the validity and feasibility of the indicators assessed, following the inclusion criteria outlined by Huanca-Marca et al. [8].

The review identified thirty valid and feasible indicators. These included body temperature; nine behavioural indicators (human–animal relationship, aggression, falling, vocalisation, slipping, panting, lying down, sitting, and turning back); corneal reflex; rhythmic breathing; and vocalisation. For the health and post-mortem category, thirteen indicators were reported: presence of entry points, hernias, body lesions, ear lesions, tail lesions, pericarditis, pneumonia, bursitis, lameness, dead animals, walking animals, and non-walking animals. Four indicators were associated with product quality (pH, bruises, body condition, and carcass weight), which are not addressed in the present review due to its specific focus.

Furthermore, the practicality of implementation in high-throughput abattoirs was considered, including ease of application, personnel requirements, equipment needs, and turnaround time for results. The current level of adoption was also assessed, ranging from proof-of-concept to full commercial availability. Table 1, Table 2 and Table 3 present the inclusion criteria for each classification: biological sample analysis, imaging and computer vision systems, and physiological and other sensors, respectively.

## 3. Results

**Figure 1:**
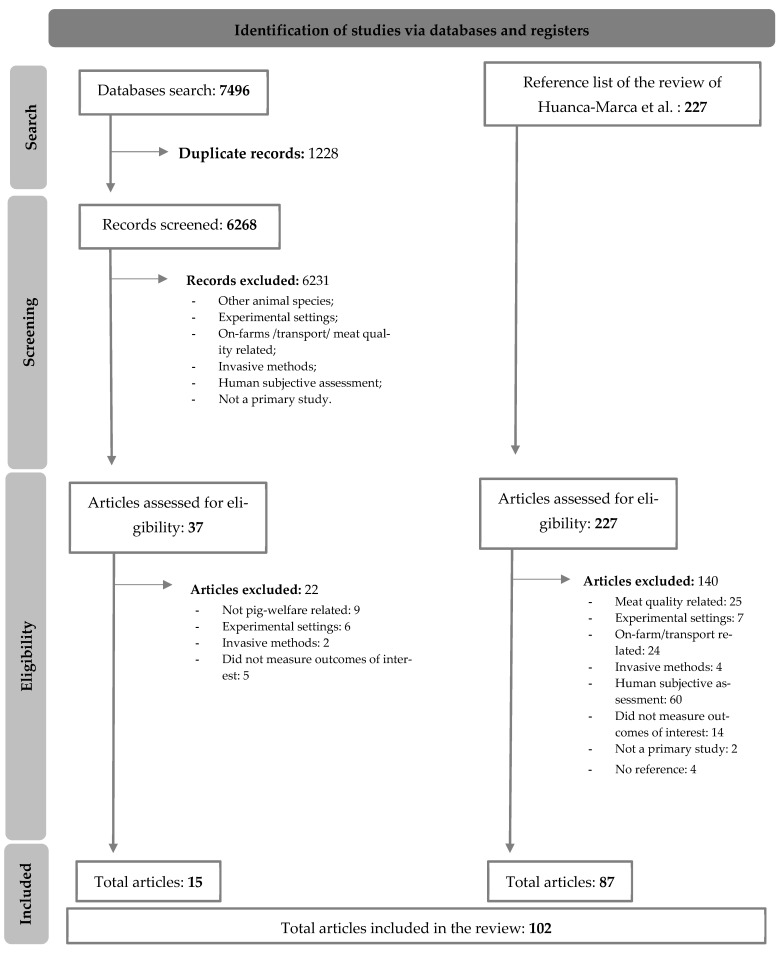
Flow diagram of the systematic literature process [8,12].

**Figure 2:**
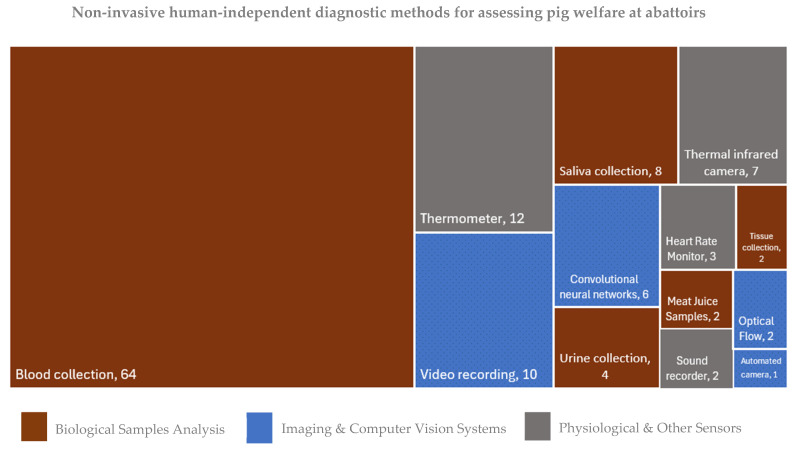
Distribution of non-invasive human-independent diagnostic methods for assessing pig welfare at abattoirs. Each rectangle’s size corresponds to a method, and its size is proportional to the number of articles citing it (n). Categories are colour-coded: orange for biological sample analysis, blue for imaging and computer vision systems, and grey for physiological and other sensors.

A flow diagram illustrating the systematic literature review process is presented in Figure 1. The initial pool of articles for this review originated from two distinct sources: the re-screening of the reference list of Huanca-Marca et al. [8] and a complementary systematic database search. From an initial pool of 7776 manuscripts, 102 articles met the eligibility criteria for inclusion. Within these, thirteen distinct non-invasive human-independent diagnostic methods were identified and categorised into three overarching groups (Figure 2). No method was classified as having both a high level of implementation and high practicality, despite the large number of studies identified. Among the methods analysed, glucose and lactate blood analysers, convolutional neural networks, and automated camera-based systems were considered highly practical for implementation due to their real-time monitoring capabilities.

The “biological sample analysis” category was the most frequently cited, encompassing 80 articles involving indicators derived from biological samples, including blood, urine, saliva, tissue collection, and meat juice sampling (Table 4). In contrast, “imaging and computer vision systems” was the least frequently cited category (*n* = 19 articles). This group includes methods that utilise visual recording and computational analysis to assess welfare and health indicators, such as video cameras, convolutional neural networks, optical flow, and automated camera-based systems (Table 5). Finally, the “physiological and other sensors” category comprises methods where indicators are derived from electrical or other specialised equipment (*n* = 24 articles), including thermal infrared cameras, thermometers, heart rate monitors, and sound recorders (Table 6).

It should be noted that some studies addressed more than one non-invasive method and were therefore included in multiple categories when different methods were applied.

## 4. Discussion

### 4.1. Biological Sample Analysis

#### 4.1.1. Blood Collection

Blood parameters obtained from exsanguinated blood can yield valuable insights into a pig’s physiological stress levels and certain health conditions. These metrics are crucial for understanding the effects of pre-slaughter handling, stunning, and overall product quality, including stress-related impacts on carcass and meat attributes. Some studies [98,102] were excluded from this category because blood was collected via puncture of the distal ear vein while the pig was still alive—an approach deemed invasive and contrary to animal welfare standards. In contrast, this review focused exclusively on blood manually collected at exsanguination and stored in appropriate tubes. However, due to the rapid pace of high-throughput slaughter lines, some sampling opportunities can be missed [24], revealing limitations in the practicality of this method under such conditions. Collected blood tubes are kept on ice until analysis in the laboratory. At this stage, the samples undergo either biochemical analysis (i.e., examination of chemical substances in plasma that indicate metabolic, endocrine, renal, hepatic, and muscular status) or haematological analysis (i.e., evaluation of blood’s cellular components and characteristics).

Huanca-Marca et al. [8] found that most physiological indicators derived from these methods had low feasibility in abattoir settings, primarily due to associated costs, time, and resource requirements. Most notably, reliance on post-collection laboratory processing precludes real-time assessment. In contrast, exsanguination blood glucose and lactate measurements are promising. They can be assessed on-site using handheld analyser devices by applying a test strip to the collected blood [15,17,18,50,106], with results typically available within ten minutes of exsanguination. More recently, an experimental study (2025) demonstrated that lactate concentrations could be obtained within approximately 13 s using a handheld meter, thereby highlighting the feasibility of near real-time monitoring [107]. Such rapid turnaround holds considerable potential for continuous welfare assessment in abattoirs. That said, it is important to recognise that biomarker levels may be influenced by numerous factors; ideally, a biomarker should correlate strongly with a specific physiological condition [68]. To ensure the reliability of blood stress indicators at the abattoir, baseline blood samples are often collected on-farm, although this incurs additional invasive procedures [23,27]. Despite these limitations, blood sampling remains widely used due to its versatility and the breadth of parameters it can assess in relation to suboptimal welfare.

#### 4.1.2. Urine Collection

Urine collection enables the analysis of corticosteroids (cortisol, cortisone), catecholamines (noradrenaline, adrenaline, dopamine), and creatinine concentrations [46,72,76,77]. Samples are typically collected directly on the slaughter line, with preservatives added to containers prior to freezing. It is crucial to account for variability in urine dilution, which can affect hormone and creatinine measurements and is often influenced by water intake [46,72,76,77]. This approach remains relatively uncommon and is primarily used to compare hormone levels with those obtained from blood or saliva samples [72]. Care must be taken to minimise the risk of contamination from the abattoir environment, which can affect the accuracy of the results.

#### 4.1.3. Saliva Collection

Saliva collection provides a minimally invasive method for analysing stress indicators in live pigs at lairage, including cortisol, oxytocin, and acute phase proteins (e.g., haptoglobin and serum amyloid A) [55,78,80,81,82]. Samples can be safely collected by non-specialist personnel using sponges attached to a metal rod for chewing, or directly by a handler using a swab [40,79]. Although concerns have been raised regarding potential anxiety from extended chewing, Jama et al. (2016) [72] found that pigs did not exhibit stress during saliva collection, as measured by other stress markers.

Collection typically requires around 60 s for adequate sponge saturation; however, Rey-Salgueiro et al. (2018) [79] reported that 75% of pigs required up to 30 min to interact with suspended ropes and deposit saliva. Despite this variability, collective saliva sampling can optimise handler time and effort. From a practical perspective, individual sampling may not always be feasible in commercial settings with large numbers of animals. In such cases, pooling samples or selecting representative subsets provides a practical compromise without substantially compromising data quality.

Proper sample preservation, such as storage in Salivette tubes under refrigeration until laboratory analysis, is essential to maintain biomarker stability and ensure accurate results [78,81]. While saliva collection shows promise as a non-invasive tool for retrospective assessment of welfare, its broader application requires careful consideration of cost-effectiveness and practicality.

#### 4.1.4. Tissue Collection

Tissue sampling is an infrequently utilised method for slaughter assessment, with only two studies identified that collected stomach, intestinal, muscle, or skin samples during the post-mortem stage [40,83]. One study [83] examined the age of skin lesions by taking biopsies from lesioned skin, which were processed and stored following specific protocols for histological, histochemical, and gene expression analyses. The second study [40] investigated porcine HSP70, a stress-inducible heat shock protein that peaks 8–10 h post-stress and remains elevated for several days, as a potential indicator of on-farm handling stress. For this purpose, stomach, intestinal, and muscle tissue samples were collected and stored at −70 °C until laboratory analysis.

Overall, this tissue collection method appears exhaustive and costly due to its laboratory requirements. Nevertheless, it offers significant advantages, including the potential to reduce human error and identify novel biomarkers for assessing welfare and health status related to farm or transport conditions.

#### 4.1.5. Meat Juice Samples

Meat juice, typically obtained by freezing and thawing muscle samples, often from the diaphragmatic muscle after carcass splitting, is routinely used at abattoirs for purposes of Salmonella controls [53]. Beyond this, it holds significant potential for assessing acute infections and chronic inflammatory conditions through the detection of acute phase proteins (APPs), such as pig major acute phase protein and haptoglobin, serving as valuable disease indicators during meat inspection [38,53]. The availability of commercial kits and competitive enzyme immunoassays for these APPs offers time and economic efficiencies for laboratories [38,53].

Despite its utility in reflecting health status, the varying release of fluid from muscle tissue—influenced by factors such as tissue blood content, pre-slaughter stress, and the presence of fascias in the tissue—presents a significant limitation [53]. This variability necessitates the development of harmonised meat juice extraction protocols, specifically addressing muscle type and sample size, to ensure that APP concentrations in meat juice accurately reflect systemic levels found in blood. Additionally, the post-mortem interval before freezing can further affect APP concentrations, and standardising this interval is important to reduce variability and improve the reliability of measurements.

### 4.2. Imaging and Computer Vision Systems

#### 4.2.1. Video Recording

Video recording is a widely utilised tool in abattoirs for various assessment purposes, including capturing animals’ behaviours during lairage and to record the effectiveness of stunning [18,22,35,65,84,85,89], after stunning (e.g., within CO_2_ stunning crates) [85,86,87], or on the processing line for carcass inspection [88]. Its primary utility lies in optimising observer time, particularly when assessment periods are extensive or when the speed of the line does not permit accurate real-time observation, analysis, or data collection [88].

However, the efficacy of video recording as a standalone method presents numerous limitations. Firstly, without integration with specialised software or automated systems, it necessitates extensive human interpretation and manual data extraction from recordings [84,86]. Secondly, video quality is frequently compromised, leading to discarded footage. Common issues include lens obstructions from dirt splashing, blurring due to high humidity (especially after scalding and dehairing), or motion blur from pig movements during lairage or in the stunning race [87,88]. These quality impairments can significantly affect observer results and inter-observer agreement [88]. Furthermore, inherent abattoir settings and conditions often restrict video recording to specific pens or small groups, limiting comprehensive coverage of entire lairage areas and posing challenges with restrictive viewing angles [85]. Recent technological innovations, such as high-humidity-resistant camera housings and improved automated analysis software, can help mitigate these limitations, allowing for more robust data collection in challenging abattoir environments.

#### 4.2.2. Convolutional Neural Networks

Computer vision systems (CVSs) have been increasingly developed and tested for automated detection of various lesions in pig carcasses, including pleurisy [91], pneumonia-like lesions [1,9], milk spot liver [10], pericarditis [10], and tail lesions [7] (Table 7). At the core of many of these systems is deep learning, particularly convolutional neural networks (CNNs). CNNs, leveraging multi-layer neural networks, are valued for their capacity to autonomously extract complex features from images, facilitating knowledge learning and enabling faster training times. However, effective training of these advanced models often relies on extensive human expert-led scoring and image annotation [108,109].

For porcine respiratory diseases, which carry a significant economic impact, CVSs have proven effective in identifying healthy lungs with high specificity for enzootic pneumonia (EP)-like lesions (99.4% [1] and 95.5% [9]). Nevertheless, CNN performance was somewhat lower for small lesions (<2 cm; sensitivity 81.3% [9]) and intermediate scores, potentially due to insufficient training data or the presence of small lesions that were not properly interpreted by experts [91]. In contrast, CNNs demonstrated very high effectiveness for pleurisy identification (sensitivity 92% [90]). In automated offal inspection, CNN models achieved higher accuracy in detecting pericarditis (93%) compared with milk spot lesions (82.2%) [10], suggesting that automated systems are more adept at classifying diffuse pathologies than focal localised ones.

Furthermore, neural networks have shown reliability in assessing tail lesions and loss directly from carcass images [7]. A study reported agreement rates between neural network classification and human observers of 74% for tail lesions and 95% for tail loss [7]. A key limitation identified was the impact of low-quality pictures, as the agreement between networks and human observers was similar to that observed between human observers themselves (80% for tail lesions, 94% for tail loss) [7].

Artificial intelligence (AI) has demonstrated superior performance in object detection and image classification tasks for pig welfare and health assessment at the abattoir [1,9,10,91]. The automated visual-only classification of pathologies in pig carcasses and offal is of significant interest due to its capacity to minimise the risk of cross-contamination associated with palpation by official veterinarians and to systematically collect consistent data during slaughter, which is particularly beneficial in high-throughput settings. This non-invasive approach not only enhances food safety by limiting microbiological risks linked to manual inspections but also ensures a standardised and unbiased evaluation of pathological conditions, enabling easy checking and analysis of farm status with the assistance of these tools. Furthermore, integrating AI technologies with real-time lesion scoring systems enables producers to track herd health trends and implement effective corrective measures.

However, a notable limitation pertains to the training of machine-learning models. Future studies must address the substantial amount of data required for models to successfully distinguish normal from pathological regions [10]. This process is inherently time-consuming, as it necessitates human experts to manually identify and annotate pathologies or areas of interest for each image.

The application of neural networks during slaughter extends to additional contexts. Pig vocalisations can be automatically classified according to their valence (positive versus negative) and context. In pig production systems, the emission of high-frequency calls—such as screams or squeals—often indicates negative emotional states like pain, frustration, or fear, thus serving as potential indicators of compromised well-being [90,105,110]. An automated emotion monitoring tool, based on a neural network and developed by Briefer et al. [90], demonstrated high accuracy for valence and context classification (91.5% and 81.5%, respectively). This tool proved robust to biological variability, despite accounting for different ages, sexes, and body sizes of the pigs from which calls were recorded. However, it was not tested in a real-time setting, even though an efficiently trained neural network can classify over 50 spectrograms per second using current smartphone hardware [90]. Conversely, the STREMODO software (prototype version) is already available as a sound analyser, capable of detecting stress screams from pigs and automatically registering their time of occurrence, duration, and intensity [111]. Only one study [27] within this systematic review actively utilised this stress monitor, reporting that high-pitched metallic or airflow sounds could lead to false-positive classifications. Given that high-pitched vocalisation is a validated and feasible indicator, already assessed by the welfare quality protocol during the final stages of driving pigs towards the stunning area [112], it remains a promising target for automated monitoring in abattoirs.

Future research should focus on expanding and diversifying training datasets to improve the robustness of CNN-based detection systems. Additionally, developing lightweight AI models and cross-platform integration of sensor and vision data could facilitate real-time monitoring and broader applicability across different abattoir settings.

#### 4.2.3. Optical Flow

Unloading at the abattoir can be a highly stressful experience for pigs, exacerbated by rapid movement encouragement from truck drivers, which can lead to tripping and stepping events [93]. To objectively identify causes of stress and physical harm, a computer vision-based approach was developed utilising optical flow (OF) and modified angular histogram (MAH) techniques, combined with a support vector machine (SVM). This computationally affordable and non-invasive method employs only a single ordinary camera to track pig movement and detect “out-of-control” situations, such as stationary animals, unusually slow movement, or excessive speed, which may indicate injury or an obstacle in the way [92,93].

OF can distinguish moving objects from backgrounds, enabling the identification and tracking of objects by analysing pixel displacements over a series of frames, making it useful for crowd analysis, as seen in pig unloading [113]. However, OF vectors alone cannot accurately capture the global movement of an individual animal when significant local movements (e.g., trotting) occur [92]. The MAH addresses this limitation by accurately identifying OF vectors that reflect the pig’s actual direction and speed [92]. To improve detection, a support vector machine identified the most critical MAH bins, successfully distinguishing moving pigs from stationary ones [93]. For detecting undesirable tripping and stepping behaviours, the integrated system achieved high classification performance, with 90% specificity for normal movements and 93.5% sensitivity for abnormal behaviours [93]. Despite this, a false alarm rate of 6.5% indicates that some normal movements can still be incorrectly classified [92].

Although computationally affordable, achieving reliable results with OF necessitates the integration of supplementary algorithms, such as MAH and SVM [92,93]. Nevertheless, this system holds significant value for real-time monitoring, enabling staff notification during unusual events such as tripping and stepping during unloading. Notably, Spain was the first country in the European Union to legislate video surveillance systems to monitor unloading areas, driving aisles, and stunning and bleeding activities [114], addressing major concerns regarding the behaviour and practices of the personnel in the meat industry.

#### 4.2.4. Automated Camera-Based System

Despite their substantial potential to provide consistent and fatigue-proof data assessment, the use of automated camera-based systems in pig abattoirs remains very limited [6]. Such systems could support individual observers responsible for evaluating welfare indicators, particularly in abattoirs slaughtering more than 1000 animals annually, as required by Regulation (EC) 1099/2009 [3]. A study by Blömke et al. [6] demonstrated the efficacy of a camera-based system for assessing ear and tail lesions, yielding strong results: for ear lesions (sensitivity, 77.0%; specificity, 96.5%; accuracy, 95.4%) and tail lesions (sensitivity, 77.8%; specificity, 99.7%; accuracy, 99.5%). Effective software development necessitates clear lesion “specifications” (i.e., defined traits); for instance, lower accuracy in ear lesion detection, compared with tail lesions, was attributed to the use of fewer algorithms, specifically lacking the incorporation of colour alongside shape and position. Beyond software development, robust camera installation is critical, as technical challenges like maintaining adequate illumination, daily calibration, preventing camera inclination (often due to cleaning processes), and managing high humidity can significantly impair image classification [6].

Nevertheless, the implementation of this system holds significant potential as a key intervention strategy in abattoirs, enabling, for instance, real-time alerts for severe tail lesions that could prompt more detailed carcass inspections by official veterinarians, thereby facilitating risk-based meat inspection and ensuring a more targeted and efficient control process [6].

### 4.3. Physiological and Other Sensors

#### 4.3.1. Thermal Infrared Camera

Thermal imaging and infrared thermography, although often used interchangeably in this review, differ in their primary focus: thermal imaging emphasises the visualisation of temperature differences, whereas infrared thermography concentrates on the quantitative analysis of thermal data. This non-invasive restraint-free method offers significant advantages for assessing pig welfare and health at the abattoir compared with traditional field-based methods [68,98]. Key benefits include minimising the risk of infection transmission during screening and the ability to integrate software for real-time data analysis [99,115].

However, accurate measurements with thermal cameras require careful consideration of several factors. It is crucial to determine the skin surface emissivity, as discrepancies can lead to temperature measurement errors of several degrees Celsius; the emissivity index for pig skin has been reported between 0.96 and 0.98 [115]. In addition, the distance between the camera and the animal, as well as the choice of measurement sites (e.g., ear base or eye region in finishing pigs), are critical [68,98]. For instance, Warriss et al. (2006) recorded three points on the inner surface of the ear and used the average for analysis [69]. Recording ambient temperature and relative humidity is also essential to minimise their potential influence on the animal’s temperature readings [68]. Previous attempts to assess the fore back anatomical region have shown limited success, likely due to factors such as dirt, hair density, and condensation on the skin, all of which can impede accurate measurement [98].

Beyond general welfare assessment, thermal imaging has shown utility in more specific applications. For example, Teixeira et al. (2020) investigated the effect of tail lesion severity on skin temperature at the base of the tail and the ear [96]. They found that increased skin temperature in both regions correlated with greater tail damage severity, suggesting that higher temperatures at the tail base indicate inflammation or potential infection [96]. Infrared imaging can also aid in predicting carcass yield loss by detecting elevated temperature regions, or “hotspots”, from dorsal images of sows [97]. Bruises, mammary infections, abscesses, and poor body condition are among the causes of trim loss in swine carcasses during post-mortem inspection [4]. Detecting these lesions with thermal imaging in live animals can therefore help anticipate substantial trim losses [97], which are associated with an increased risk of contamination along the processing line.

#### 4.3.2. Thermometer

Physiological indicators, such as respiratory rate and rectal temperature, are crucial for assessing heat-stressed pigs [116]. Pig body temperature is typically measured using digital, mercury, or infrared thermometers at various sites, including the rectum, ear, or skin [35,50,51,68,69,71,94,99,100,101,102,103]. Rectal temperature, assessed by inserting a digital or mercury thermometer directly into the rectum until its bulb contacts the rectal mucosa [94], requires direct animal contact and is often time-consuming. However, the use of mercury thermometers has been prohibited in the European Union since 2009 due to their toxic effects in case of breakage [117]. Ear temperature, measured with a digital thermometer, can be assessed more rapidly, typically within approximately one second per animal [102]. Surface temperature can be measured using a laser infrared thermometer, with readings commonly taken at the withers, middle ribs, or midline [100,101].

Despite these approaches, traditional temperature measurement remains labour-intensive. This represents a significant challenge for large-scale applications in commercial abattoirs, particularly given the high speed of the slaughter line, which can impede proper individual assessment. Manual measurement and data recording are difficult to implement on a large scale, even with restraint equipment such as a “pipe” during lairage [94], or considering the speed of the line after stunning [68,71]. Non-contact temperature measurement methods, such as infrared thermography, have gained prominence, although rectal temperature is generally considered a more reliable indicator than skin temperature [116].

Furthermore, while skin, ear, and rectal temperatures have been used to estimate core body temperature, blood flowing in major arteries near the heart provides a more accurate estimate [35,69,99]. Exsanguinated blood can be measured using a digital thermometer or a laser device, and blood temperatures have been shown to correlate with pen temperatures and to respond sensitively to changes in environmental conditions [99].

#### 4.3.3. Heart Rate Monitor

Heart rate monitoring has been studied as an indicator of physiological stress in pigs from farm loading through to unloading at the abattoir [36,55,62,104]. Accurate assessment requires specialised equipment and controlled conditions. The heart rate monitor must be secured around the pig’s chest, typically on the left side of the thorax, at least 24 h prior to data collection to allow for animal habituation and to ensure stable readings unaffected by interactions with other animals. This portable non-invasive system detects and stores electrocardiograms (ECGs) for subsequent analysis of inter-beat intervals, although human interpretation is ultimately required [36,55,118]. Consequently, this technique often proves impractical for field conditions or in high-throughput abattoir settings, and its use may also conflict with biosecurity protocols in certain slaughter plants. In contrast, the photoplethysmography (PPG) technique, widely used in humans, is currently being investigated as a cost-effective method capable of extracting reliable heart rate signals. Research is also focused on developing real-time monitoring algorithms to derive pig heart rate using this technology [118].

#### 4.3.4. Sound Recorder

As previously mentioned, pig vocalisations are associated with stressful events. Common causes of animal welfare problems in abattoir design, such as electric prodding, slick floors, and narrow aisles where pigs cannot move side-by-side, frequently trigger high-frequency calls [110]. To enable objective assessment of stress-related vocalisations and thereby bypass subjective human interpretation, voice recorders are commonly positioned on the unloading truck ramp and within lairage pens to capture different types of vocalisations [27,105]. This approach differs methodologically from neural network-based classification, as it involves direct audio acquisition rather than automated analysis of audio data. However, a study by Støier et al. [105], which employed a wireless microphone, encountered challenges with precise microphone placement, leading to sound overloading that prevented accurate analysis of the recorded vocalisation intensity. Subsequently, vocalisations can be manually [105] or automatically classified [27,90] according to their valence and context.

## 5. Overall Discussion

This systematic review identified thirteen non-invasive human-independent methods for monitoring pig welfare and health at abattoirs, grouped into three categories: biological sample analysis, imaging and computer vision systems, and physiological and other sensors. Each presented distinct advantages and limitations.

Biological sample analysis methods are the most frequently cited and provide reliable physiological indicators. However, while identifying stress and health-related indicators is essential, the effectiveness of these methods depends on the timely implementation of corrective actions. Merely diagnosing welfare issues without prompt intervention may have a limited impact, particularly for failures in pre-slaughter handling that rely on laboratory results or subsequent human analysis. Furthermore, these methods differ in labour intensity and time requirements; techniques such as blood, urine, saliva, or tissue collection are more time-consuming and require strict biosecurity measures compared with automated imaging or sound analysis systems.

Imaging and computer vision systems offer real-time monitoring and substantial potential to improve standardisation and reduce observer fatigue; however, technical limitations remain. Their effectiveness depends on algorithm performance, camera setup, lighting, and environmental conditions, necessitating calibration and maintenance. Ethical considerations, including responsible AI use and data transparency, should guide the adoption of these technologies in commercial abattoirs.

Physiological and other sensors can promptly detect stress or welfare issues. While less labour-intensive, their accuracy and throughput can be affected by sensor placement and environmental factors.

Across all categories, no method fully combines high practicality and ease of implementation, though blood analysers, automated cameras, and neural networks show promise for real-time results, enabling immediate corrective actions.

Limitations of the present study include the possibility that not all relevant articles were identified, particularly studies in the grey literature or non-English/Portuguese/Spanish publications. Additionally, some methods may overlap in the indicators they measure, complicating direct comparisons between techniques.

## 6. Conclusions

Pig welfare and health assessment at slaughter can be evaluated with non-invasive human-free tools, ranging from thermal imaging and salivary biomarkers to AI-driven computer vision systems. While their routine application remains limited in high-throughput settings, glucose and lactate blood analysers, convolutional neural networks, and automated camera-based systems are the most promising methods for ensuring practical and real-time monitoring on the slaughter line.

Furthermore, the economic viability of non-automated methods must be carefully evaluated to determine whether their implementation is viable for daily and routine use in commercial settings. Ultimately, only with valid and consistent data, supported by standardisation protocols and rigorous validation studies, can we ensure accurate assessment, regulatory acceptance, and continuous improvement in animal welfare and food safety.

## Figures and Tables

**Table 1:** Inclusion criteria for classification of the diagnostic methods of biological sample analysis.

Classification	Validity and Feasibility (V&F) of Indicators	Practicality of Implementation	Level of Implementation
High	Uses ≥1 of the 29 high V&F indicators identified by Huanca-Marca et al. [8]	Requires personnel, but the method is fast, easy to perform at the abattoir, and results are available immediately	Commercially available or widely validated
Medium	Uses V&F indicators, but requires contextual validation or a less standardised application	Requires personnel to collect samples and send them to a laboratory; portable in some cases but with added complexity	Tested prototype or limited commercial availability
Low	Uses indicators with low V&F (Huanca-Marca et al. [8])	Requires personnel, complex protocols, and lab-based analysis with significant delays; no automation potential	Still at the proof-of-concept stage or tested in very few studies

**Table 2:** Inclusion criteria for classification of the diagnostic methods of imaging and computer vision systems.

Classification	Validity and Feasibility (V&F) of Indicators	Practicality of Implementation	Level of Implementation
High	Uses ≥1 of the 29 high V&F indicators identified by Huanca-Marca et al. [8]	Fully automated real-time analysis with no human input required	Commercially available or widely validated
Medium	Uses V&F indicators but requires contextual validation or has a less standardised application	Semi-automated systems or methods requiring human supervision	Tested prototype or limited commercial availability
Low	Uses indicators with low V&F (Huanca-Marca et al. [8])	Manual data analysis or human-dependent image review	Still at the proof-of-concept stage or tested in very few studies

**Table 3:** Inclusion criteria for classification of the diagnostic methods of physiological and other sensors.

Classification	Validity and Feasibility (V&F) of Indicators	Practicality of Implementation	Level of Implementation
High	Uses ≥1 of the 29 high V&F indicators identified by Huanca-Marca et al. [8]	Automatic, continuous, and real-time data capture with minimal or no human intervention	Commercially available or widely validated
Medium	Uses V&F indicators, but requires contextual validation or a less standardised application	Requires some manual setup, calibration, or human involvement for data collection or interpretation	Tested prototype or limited commercial availability
Low	Uses indicators with low V&F (Huanca-Marca et al. [8])	Complex equipment; full operator involvement; not feasible for high-throughput abattoirs	Still at the proof-of-concept stage or tested in very few studies

**Table 4:** Non-invasive human-independent diagnostic methods based on biological sample analysis for pig welfare assessment at abattoirs.

Method	Articles	Indicators	Validity and Feasibility	Practicality of Implementation	Level of Implementation
Blood Collection	[13,14,15,16,17,18,19,20,21,22,23,24,25,26,27,28,29,30,31,32,33,34,35,36,37,38,39,40,41,42,43,44,45,46,47,48,49,50,51,52,53,54,55,56,57,58,59,60,61,62,63,64,65,66,67,68,69,70,71,72,73,74,75]	Acute phase proteins ACTH Amylase Blood pH Corticosterone Cortisol Creatine kinase Creatine phosphokinase Electrolytes Glucose Haematocrit Insulin-like growth factor Lactate Lactate dehydrogenase Non-esterified fatty acids Testosterone and oestradiol Total serum protein Gasometria parameters Other haematological parameters Other biochemical parameters Genetic analysis (gDNA, ryr-1)	Medium Medium NR^1^ NR^1^ Medium Medium Medium Medium NR^1^ Medium NR^1^ NR^1^ Medium Medium Medium NR^1^ NR^1^ NR^1^ NR^1^ NR^1^ NR^1^	Medium-High	High
Urine Collection	[46,72,76,77]	Cortisol Cortisone Catecholamines Creatinine	NR^1^ Low Low Medium	Medium	Low
Saliva Collection	[40,55,72,78,79,80,81,82]	Acute phase proteins Adenosine deaminase Alpha-amylase Butyrylcholinesterase Calprotectin Cortisol Lactate dehydrogenase Oxytocin Total esterase activity	Medium NR^1^ Medium NR^1^ NR^1^ NR^1^ Medium NR^1^ NR^1^	Medium	High
Tissue Collection	[40,83]	Skin lesions Heat shock protein HSP70	High NR^1^	Medium	Low
Meat Juice Samples	[38,53]	Acute phase proteins	Medium	Medium	Medium

NR^1^: not reported.

**Table 5:** Non-invasive human-independent diagnostic methods using imaging and computer vision systems for pig welfare assessment at abattoirs.

Method	Articles	Indicators	Validity and Feasibility	Practicality of Implementation	Level of Implementation
Video Recording	[18,30,35,65,84,85,86,87,88,89]	Pre-slaughter behaviours Slaughter behaviours Tail lesions	Medium-High Medium-High High	Low	High
Convolutional Neural Networks	[1,7,9,10,90,91]	Vocalisation Tail lesions Pleurisy Pneumonia Milk spot liver Pericarditis	High High Medium High Medium High	High	High
Optical Flow	[92,93]	Pig movement	NR^1^	Medium	Low
Automated Camera-Based System	[6]	Ear and tail lesions	High	High	Low

NR^1^: not reported.

**Table 6:** Non-invasive human-independent diagnostic methods using physiological and other sensors for pig welfare assessment at abattoirs.

Method	Articles	Indicators	Validity and Feasibility	Practicality of Implementation	Level of Implementation
Thermal Infrared Camera	[61,68,94,95,96,97,98]	Skin temperature Ocular temperature	High Medium	Medium	High
Thermometer	[35,50,51,68,69,71,94,99,100,101,102,103]	Skin temperature Body temperature Rectal temperature Blood temperature	High High NR^1^ NR^1^	Low	High
Heart Rate Monitor	[36,55,104]	Heart Rate	Medium	Low	Low
Sound Recorder	[27,105]	Vocalisation	High	Medium	Medium

NR^1^: not reported.

**Table 7:** Performance of computer vision systems in detecting lesions in pig carcasses at abattoirs.

Reference	Organ Evaluated	Specific Lesions	CVS ^1^ Performance
[91]	Lungs (pleura)	Pleurisy	SE ^2^: 92.0% SP ^3^: 96.0%
[10]	Liver Heart	Milk spots Pericarditis	SE: 77.3%, SP: 86.4% (milk spots) SE: 92.6%, SP: 93.4% (pericarditis)
[1]	Lungs	Enzootic pneumonia-like lesions	SE: 81.3% (lesion size <2% of the entire lung surface) SE: 100% (lesion size between 2 and 5% of the entire lung surface) SE: 100% (lesion size between 5 and 10% of the entire lung surface) SE: 100% (lesions >10% of the entire lung surface) SP: 99.4%
[9]	Lungs	Enzootic pneumonia-like lesions	SE: 85.0% SP: 95.5%
[7]	Skin	Tail lesions	SE; SP: not reported Agreement for tail lesions: 0.74 Agreement for tail loss: 0.94

^1^ CVS—computer vision systems; ^2^ SE—sensitivity; ^3^ SP—specificity.
